# Determining the Anticancer Activity of Sphingosine Kinase Inhibitors Containing Heteroatoms in Their Tail Structure

**DOI:** 10.3390/pharmaceutics14010157

**Published:** 2022-01-10

**Authors:** Jitendra Shrestha, Seong Woong Kim, Su-Bin Kim, Yoon Sin Oh, Sung Hwan Ki, Taeho Lee, Sang-Bum Kim, Taeuk Park, Dong Jae Baek, Eun-Young Park

**Affiliations:** 1 College of Pharmacy, Mokpo National University, Muan-gun 58554, Korea; shresthasimon2011@gmail.com (J.S.); 76pey@naver.com (S.W.K.); rlatnqls0801@naver.com (S.-B.K.); 2 Department of Food and Nutrition, Eulji University, Seongnam 13135, Korea; ysoh@eulji.ac.kr; 3 College of Pharmacy, Chosun University, Gwangju 61452, Korea; shki@chosun.ac.kr; 4 College of Pharmacy, Research Institute of Pharmaceutical Sciences, Kyungpook National University, Daegu 41566, Korea; tlee@knu.ac.kr; 5 New Drug Development Center, Daegu-Gyeongbuk Medical Innovation Foundation, Daegu 41061, Korea; ksb2014@dgmif.re.kr; 6 Laboratory Animal Center, Daegu-Gyeongbuk Medical Innovation Foundation, Daegu 41061, Korea; tw3000@dgmif.re.kr

**Keywords:** SK inhibitor, colorectal cancer, S1P, derivative, anticancer agent, PP2A

## Abstract

Sphingosine kinase (SK) enzyme, a central player of sphingolipid rheostat, catalyzes the phosphorylation of sphingosine to the bioactive lipid mediator sphingosine 1 phosphate (S1P), which regulates cancer cell proliferation, migration, differentiation, and angiogenesis through its extracellular five G protein-coupled S1P receptors (S1PR_1–5_). Recently, several research studies on SK inhibitors have taken place in order use them for the development of novel anticancer-targeted therapy. In this study, we designed and synthesized analog derivatives of known SK1 inhibitors, namely RB005 and PF-543, by introducing heteroatoms at their tail structure, as well as investigated their anticancer activities and pharmacokinetic parameters in vitro. Compounds **1**–**20** of RB005 and PF-543 derivatives containing an aliphatic chain or a tail structure of benzenesulfonyl were synthesized. All compounds of set 1 (**1**–**10**) effectively reduced cell viability in both HT29 and HCT116 cells, whereas set 2 derivatives (**11**–**20**) showed poor anticancer effect. Compound **10**, having the highest cytotoxic effect (48 h, HT29 IC_50_ = 6.223 µM, HCT116 IC_50_ = 8.694 µM), induced HT29 and HCT116 cell death in a concentration-dependent manner through the mitochondrial apoptotic pathway, which was demonstrated by increased annexin V-FITC level, and increased apoptotic marker cleaved caspase-3 and cleaved PARP. Compound **10** inhibited SK1 by 20%, and, thus, the S1P level decreased by 42%. Unlike the apoptosis efficacy, the SK1 inhibitory effect and selectivity of the PF-543 derivative were superior to that of the RB005 analog. As a result, compounds with an aliphatic chain tail exhibited stronger apoptotic effects. However, this ability was not proportional to the degree of SK inhibition. Compound **10** increased the protein phosphatase 2A (PP2A) activity (1.73 fold) similar to FTY720 (1.65 fold) and RB005 (1.59 fold), whereas compounds **11** and **13** had no effect on PP2A activation. Since the PP2A activity increased in compounds with an aliphatic chain tail, it can be suggested that PP2A activation has an important effect on anticancer and SK inhibitory activities.

## 1. Introduction

Sphingosine kinase (SK), an evolutionarily conserved lipid kinase, transforms sphingosine into an active lipid mediator sphingosine-1-phosphate (S1P) [1]. S1P regulates various biological processes such as cell proliferation, migration, differentiation, and angiogenesis through five G protein-coupled S1P receptors (S1PR) binding [2]. SK has two isotypes, i.e., SK1 and 2, and is present in specific cellular locations. Both the isotypes have potential oncogenic characteristics [3,4] upon overexpression and promote cancer cell proliferation, angiogenesis, metastasis, and resistance against radiation and various chemotherapeutic agents [5,6]. For this reason, SK1 and 2 serve as novel targets for the development of anticancer agents. Various SK1, SK2, and S1PR inhibitors have been developed and tested in several preclinical tumor models.

**Figure 1:**
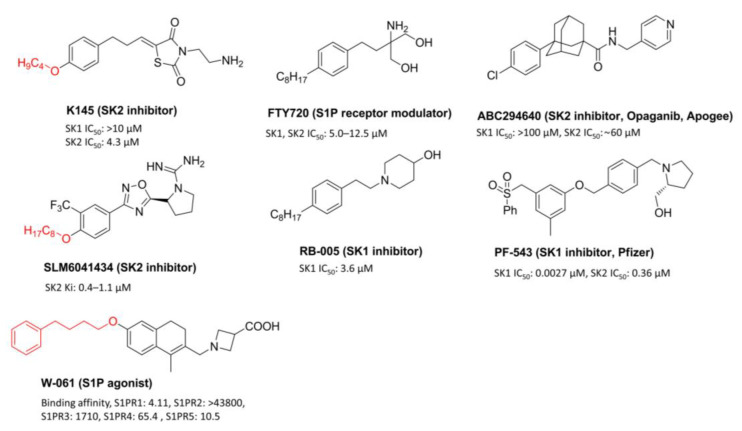
Known sphingolipid regulators containing a heteroatom tail, FTY720 and SK inhibitors.

FTY720 (Gilenya, Novartis), a modulator of the S1P receptor, suppresses immunity and has been developed to treat multiple sclerosis (Figure 1). FTY720 also suppresses SK1 and activates PP2A to show a strong anticancer effect against various cancer cells in vitro and in vivo. For this reason, various sphingolipid modulators with FTY720′s structure have been developed. However, the synthesis of FTY720 derivatives is challenging because, structurally, its polar head group and hydrophobic tail are directly connected to the aromatic backbone. Considering this difficulty, several research groups have developed various sphingolipid modulators with a tail structure containing heteroatoms. RB005 is an analog of FTY720 and is a compound that selectively inhibits SK1. Previously, we synthesized several FTY720 analogs, of which compound **7** exhibited PP2A-activating properties and anticancer effects against colorectal and prostate cancer cells [7]. A sphingolipid modulator, K145 [8], having the FTY720 structure, was developed at the University of Virginia. K145 is an SK2 inhibitor that is undergoing development to serve as a leukemia treatment substance. SLM6041434 selectively inhibits [9] SK2, and W-061, with a dihydronaphthalene structure, is an S1P agonist [10] (Figure 1). In these analogs, a polar complex head group and the aliphatic tail of FTY720 are connected with a heteroatom. A tail structure connected by a heteroatom makes it easy to introduce various functional groups synthetically. In addition, this structure reduces solubility in water due to its long hydrophobic tail.

ABC294640 (Opaganib, Apogee Biotech.) is a substance that exhibits anticancer activity by selectively inhibiting SK2 and is currently undergoing phase 2 clinical trials in various solid cancers (Figure 1) [8]. PF-543, developed by Pfizer, is the strongest SK1 inhibitor [9]. PF-543 demonstrates poor anticancer activity in several cancer cells, despite its strong SK1 inhibitory effect (IC_50_ = 3.4 nM, Figure 1) [10]. Although many SK1 inhibitors are being developed, PF-543 still shows the strongest SK1 inhibitory effect, and, for this reason, the development of PF-543 derivatives is required. Pfizer reported the synthesis and SK inhibitory effects of PF-543 derivatives in 2017 [11]. These results revealed that PF-543 still exhibited the highest SK1 inhibitory effect, and the derivatives modified with the benzenesulfonyl tail structure of PF-543 showed a lowered SK1 inhibitory effect. These results showed that the benzenesulfonyl tail structure plays an important structural role in the SK1 inhibitory effect. We synthesized a derivative by introducing an aliphatic chain, which was similar to that of FTY720, in the structure of PF-543 and compared SK inhibitory effect and anticancer activity between PF-543 and its derivative [12]. It was revealed that the synthesized PF-543 derivative showed SK1 inhibitory and anticancer effects similar to those of PF-543. However, this synthesis method using Sonogashira coupling is not suitable for synthesizing various derivatives. We designed and synthesized novel RB005 and PF-543 analogs by introducing heteroatoms in the tail using a convenient synthesis method. Through this, we tried to confirm the effect of a heteroatom tail on SK inhibitory effect and anticancer activity. In addition, we investigated how the aliphatic tail and benzenesulfonyl tail structures affect anticancer activity using colorectal cancer cells.

## 2. Materials and Methods

### 2.1. Chemistry

#### 2.1.1. Synthesis in General

Reagents and solvents used for the reaction were purchased commercially. Silica gel used for column chromatography was grade 60 (230–400 mesh). ^1^H-NMR and ^13^C-NMR were measured using JEOL ECZ500R (JEOL Co., Tokyo, Japan) at 500 and 125 MHz, respectively, and a deuterated solvent was used. High-resolution mass spectra were measured using an Agilent Technologies G6520A Q-TOF mass spectrometer (Santa Clara, CA, USA) using electrospray ionization (ESI).

#### 2.1.2. Chemical Synthesis (**12**–**16**)

##### 4-(2-(Trityloxy)ethyl)phenol (**12**)

To a solution of 4-(2-hydroxyethyl)phenol (1 g, 0.007 mol) and 4-dimethylaminopyridine (DMAP) (88 mg, 0.72 mmol) in CH_2_Cl_2_/pyridine (1/2, 100 mL) was added trityl chloride (2 g, 0.007 mol) at rt. After the reaction, the mixture was stirred at room temperature for 1 d, and the mixture was extracted with EtOAc. The combined organic phases were washed with brine, dried and concentrated. Flash column chromatography with *n*-hexane:EtOAc (3:1 (*v*/*v*)) as the eluent gave compound **12** (1.49 g, 56%): ^1^H NMR (500 MHz, CDCl_3_) δ 7.41–7.36 (m, 6H), 7.32–7.25 (m, 6H), 7.24–7.20 (m, 3H), 7.05 (d, *J* = 8.7 Hz, 2H), 6.75 (d, *J* = 8.7 Hz, 2H), 3.26 (t, *J* = 7.1 Hz, 2H), 2.82 (t, *J* = 7.2 Hz, 2H); ^13^C NMR (125 MHz, CDCl_3_) δ 185.3, 154.1, 149.2, 143.3, 185.3, 185.3, 257.3, 127.4, 127.0, 115.2, 65.3, 35.9; ESI-HRMS (M+H)^+^ *m/z* calcd for C_27_H_25_O_2_ 381.1855, found 381.1870.

##### (4-(Heptyloxy)phenethoxy)methanetriyl)tribenzene (**13**)

Compound **12** (1 g, 0.0026 mol) was dissolved in DMF (100 mL), K_2_CO_3_ (1.08 g, 0.0078 mol) and 1-bromoheptane (0.83 mL, 0.0053 mol) were added, and the mixture was stirred at 80 °C for 12 h. Water was added to stop the reaction, and it was concentrated under reduced pressure after EtOAc extraction and MgSO_4_ drying. The mixture was separated by column chromatography (*n*-hexane:EtOAc = 5:1) to give compound **13** (0.94 g, 75%): ^1^H NMR (500 MHz, CDCl_3_) δ 7.39–7.34 (m, 6H), 7.27–7.23 (m, 6H), 7.22–7.17 (m, 3H), 7.08 (d, *J* = 8.6 Hz, 2H), 6.80 (d, *J* = 8.7 Hz, 2H), 3.91 (t, *J* = 6.6 Hz, 2H), 3.23 (t, *J* = 7.0 Hz, 2H), 2.82 (t, *J* = 7.0 Hz, 2H), 1.43 (ddd, *J* = 12.1, 9.2, 6.9 Hz, 2H), 1.38–1.24 (m, 6H), 0.88 (t, *J* = 6.9 Hz, 3H); ^13^C NMR (125 MHz, CDCl_3_) δ 157.7, 144.4, 131.2, 130.2, 128.8, 128.0, 127.8, 126.9, 114.4, 86.6, 68.1, 65.3, 35.9, 31.9, 31.6, 29.4, 29.2, 29.0, 26.1, 22.7, 14.2; ESI-HRMS (M + H)^+^*m/z* calcd for C_34_H_39_O_2_ 479.2950, found 479.2915.

##### 4-(Heptyloxy)phenethyl 4-methylbenzenesulfonate (**15**)

Compound **13** (500 mg, 1.044 mol) was dissolved in MeOH/CH_2_Cl_2_ (5/1, 50 mL), conc. HCl (1 mL) was added thereto, and the mixture was stirred at 50 °C for 5 days. Water was added to stop the reaction, and it was concentrated under reduced pressure after EtOAc extraction and MgSO_4_ drying. The resulting mixture **14** was dissolved in CH_2_Cl_2_ (50 mL) without purification, and triethylamine (0.73 mL, 0.005 mol) and *p*-toluenesulfonyl chloride (299 mg, 1.567 mmol) were added thereto and stirred at room temperature for 12 h. The reaction was terminated with water and EtOAc, and the organic layer was dried over MgSO_4_ and concentrated under reduced pressure. The resulting mixture was separated by column chromatography (*n*-hexane:EtOAc = 5:1) to give compound **15** (171 mg, 42%): ^1^H NMR (500 MHz, CDCl_3_) δ 7.68 (d, *J* = 8.3 Hz, 2H), 7.27 (d, *J* = 8.6 Hz, 2H), 7.11 (d, *J* = 8.7 Hz, 2H), 7.00 (d, *J* = 8.7 Hz, 2H), 4.15 (t, *J* = 7.1 Hz, 2H), 3.92 (t, *J* = 6.6 Hz, 2H), 2.87 (t, *J* = 7.1 Hz, 2H), 2.42 (s, 3H), 1.76 (tt, *J* = 7.0, 3.6 Hz, 2H), 1.44 (dt, *J* = 15.0, 7.0 Hz, 2H), 1.39–1.25 (m, 6H), 0.89 (t, *J* = 6.7 Hz, 3H); ESI-HRMS (M + H)^+^ *m*/*z* calcd for C_22_H_31_O_4_S 391.1943, found 391.1992.

##### 4-(2-((Phenylsulfonyl)oxy)ethyl)phenyl benzenesulfonate (**16**)

After 4-(2-hydroxyethyl)phenol (1 g, 0.0072 mol) was dissolved in 1,2-dichloromethane (40 mL), triethylamine (5 mL, 0.036 mol) and benzenesulfonyl chloride (3.8 g, 0.022 mol) were added at rt. It was stirred for 12 h. Water and EtOAc were added to the reaction mixture, and the organic layer was dried with MgSO_4_. The resulting mixture was separated by column chromatography (*n*-hexane:EtOAc = 10:1) to give compound **16** (2.7 g, 89%): ^1^H NMR (500 MHz, CDCl_3_) δ 7.78 (ddt, *J* = 23.5, 8.4, 1.3 Hz, 4H), 7.63 (dddd, *J* = 8.1, 7.3, 5.1, 1.3 Hz, 2H), 7.54–7.45 (m, 4H), 7.01 (dd, *J* = 8.4, 1.2 Hz, 2H), 6.84 (dd, *J* = 8.6, 1.5 Hz, 2H), 4.18 (td, *J* = 6.7, 1.5 Hz, 2H), 2.90 (t, *J* = 6.7 Hz, 2H); ^13^C NMR (125 MHz, CDCl_3_) δ 148.5, 135.9, 135.6, 135.4, 134.4, 133.9, 130.2, 129.3, 128.5, 127.8, 122.6, 70.4, 34.7; ESI-HRMS (M + H)^+^ *m*/*z* calcd for C_20_H_19_O_6_S_2_ 419.0623, found 419.0611.

#### 2.1.3. Synthesis of Compounds **1**–**20**

To a solution of **15** (50 mg, 0.13 mmol) or **16** (50 mg, 0.12 mmol) in 7 mL of acetonitrile was added cyclic amine (0.38 mmol). The reaction mixture was stirred at 50 °C for 12 h and concentrated. Purification by silica gel chromatography, eluting with CH_2_Cl_2_/MeOH (10:1), gave compounds **1**–**20**. Cyclic amine ((*R*)-(−)-prolinol (**1**, **11**), piperidine (**2**, **12**), 4-hydroxypiperidine (**3**, **13**), morpholine (**4**, **14**), (*S*)-(+)-prolinol (**5**, **15**), 4-piperidinemethanol (**6**, **16**), 1-methylpiperidine (**7**, **17**), 3-piperidinemethanol (**8**, **18**), piperazine (**9**, **19**), 1-(2-hydroxyethyl)piperazine (**10**, **20**)) was used for each reaction.

(*R*)-(1-(4-(Heptyloxy)phenethyl)pyrrolidin-2-yl)methanol (**1**): ^1^H NMR (500 MHz, CDCl_3_) δ 7.11 (d, *J* = 8.6 Hz, 2H), 6.81 (d, *J* = 8.6 Hz, 2H), 3.93 (dd, *J* = 13.1, 3.0 Hz, 1H), 3.89 (t, *J* = 6.6 Hz, 2H), 3.83 (dd, *J* = 13.1, 6.5 Hz, 1H), 3.76–3.70 (m, 1H), 3.52–3.41 (m, 1H), 3.39–3.34 (m, 1H), 3.20 (ddd, *J* = 14.6, 10.7, 7.7 Hz, 1H), 3.06–2.96 (m, 2H), 2.85–2.78 (m, 1H), 2.14–1.93 (m, 4H), 1.78–1.69 (m, 2H), 1.42 (dt, *J* = 15.0, 6.9 Hz, 2H), 1.36–1.23 (m, 6H), 0.87 (t, *J* = 6.9 Hz, 3H); ^13^C NMR (125 MHz, CDCl_3_) δ 158.3, 129.8, 115.0, 68.1, 60.9, 54.8, 31.9, 31.5, 29.3, 29.1, 26.5, 26.1, 24.1, 22.7, 14.2; ESI-HRMS (M + H)^+^*m/z* calcd for C_20_H_34_NO_2_ 320.2590, found 320.2528.

1-(4-(Heptyloxy)phenethyl)piperidine (**2**): ^1^H NMR (500 MHz, CDCl_3_) δ 7.10 (d, *J* = 8.6 Hz, 2H), 6.79 (d, *J* = 8.7 Hz, 2H), 3.88 (t, *J* = 6.6 Hz, 2H), 3.11–3.04 (m, 2H), 3.01–2.94 (m, 2H), 2.01–1.90 (m, 2H), 1.73 (dq, *J* = 13.3, 6.6 Hz, 2H), 1.64–1.52 (m, 2H), 1.44–1.37 (m, 2H), 1.35–1.18 (m, 8H), 0.86 (t, *J* = 6.9 Hz, 3H); ^13^C NMR (125 MHz, CDCl_3_) δ 158.2, 129.8, 126.0, 114.9, 68.1, 59.5, 53.6, 31.9, 30.0, 29.3, 29.1, 26.1, 23.3, 22.6, 14.1; ESI-HRMS (M + H)^+^*m/z* calcd for C_20_H_34_NO 304.2640, found 304.2639.

1-(4-(Heptyloxy)phenethyl)piperidin-4-ol (**3**): ^1^H NMR (500 MHz, CDCl_3_) δ 6.99 (d, *J* = 8.6 Hz, 2H), 6.74 (d, *J* = 8.7 Hz, 2H), 4.00–3.91 (m, 1H), 3.86 (t, *J* = 6.6 Hz, 2H), 3.19 (t, *J* = 9.1 Hz, 2H), 2.96–2.87 (m, 6H), 2.14–2.07 (m, 2H), 1.90–1.84 (m, 2H), 1.72 (dq, *J* = 13.3, 6.6 Hz, 2H), 1.40 (dq, *J* = 9.1, 6.8 Hz, 2H), 1.35–1.22 (m, 6H), 0.86 (t, *J* = 6.9 Hz, 3H); ^13^C NMR (125 MHz, CDCl_3_) δ 158.2, 129.8, 129.1, 114.8, 68.1, 58.9, 31.9, 29.4, 29.2, 26.2, 22.8, 21.4, 14.2; ESI-HRMS (M + H)^+^*m/z* calcd for C_20_H_34_NO_2_ 320.2590, found 320.2591.

4-(4-(Heptyloxy)phenethyl)morpholine (**4**): ^1^H NMR (500 MHz, CDCl_3_) δ 7.09 (d, *J* = 8.6 Hz, 2H), 6.81 (d, *J* = 8.6 Hz, 2H), 3.91 (t, *J* = 6.6 Hz, 2H), 3.80–3.72 (m, 4H), 2.76 (dd, *J* = 9.9, 6.5 Hz, 2H), 2.64–2.48 (m, 6H), 1.75 (dt, *J* = 14.6, 6.6 Hz, 2H), 1.43 (ddd, *J* = 12.1, 9.1, 6.9 Hz, 2H), 1.38–1.23 (m, 6H), 0.88 (t, *J* = 6.9 Hz, 3H); ^13^C NMR (125 MHz, CDCl_3_) δ 157.7, 129.6, 128.0, 114.6, 68.1, 66.9, 61.1, 53.7, 31.9, 29.4, 29.2, 26.1, 22.7, 14.2; ESI-HRMS (M + H)^+^*m/z* calcd for C_19_H_32_NO_2_ 306.2433, found 306.2478.

(*S*)-(1-(4-(Heptyloxy)phenethyl)pyrrolidin-2-yl)methanol (**5**): ^1^H NMR (500 MHz, CDCl_3_) δ 7.13 (d, *J* = 8.6 Hz, 2H), 6.83 (d, *J* = 8.6 Hz, 2H), 3.91 (t, *J* = 6.6 Hz, 2H), 3.86–3.79 (m, 1H), 3.47 (ddd, *J* = 11.1, 9.2, 5.7 Hz, 2H), 3.39–3.30 (m, 1H), 3.17–3.02 (m, 2H), 2.87 (ddd, *J* = 14.5, 7.0, 4.2 Hz, 1H), 2.26–2.14 (m, 2H), 2.11–1.99 (m, 4H), 1.75 (dt, *J* = 14.5, 6.6 Hz, 2H), 1.47–1.38 (m, 2H), 1.37–1.17 (m, 6H), 0.88 (t, *J* = 6.7 Hz, 3H); ^13^C NMR (125 MHz, CDCl_3_) δ 158.5, 129.8, 127.7, 115.1, 71.4, 68.2, 60.8, 58.7, 54.8, 31.9, 31.0, 29.8, 29.3, 29.1, 26.4, 26.1, 24.4, 22.7, 14.2; ESI-HRMS (M + H)^+^*m/z* calcd for C_20_H_34_NO_2_ 320.2590, found 320.2531.

(1-(4-(Heptyloxy)phenethyl)piperidin-4-yl)methanol (**6**): ^1^H NMR (500 MHz, CDCl_3_) δ 7.07 (d, *J* = 8.6 Hz, 2H), 6.79 (d, *J* = 8.7 Hz, 2H), 3.89 (t, *J* = 6.6 Hz, 2H), 3.70 –3.60 (m, 2H), 3.53 (d, *J* = 5.9 Hz, 2H), 3.13–3.03 (m, 4H), 2.79–2.61 (m, 2H), 2.03–1.86 (m, 4H), 1.74 (dt, *J* = 14.6, 6.6 Hz, 3H), 1.41 (tt, *J* = 9.3, 6.8 Hz, 2H), 1.37–1.22 (m, 6H), 0.87 (t, *J* = 7.0 Hz, 3H); ^13^C NMR (125 MHz, CDCl_3_) δ 158.4, 129.8, 126.0, 115.0, 68.2, 65.0, 61.2, 48.1, 34.2, 31.9, 29.3, 29.1, 26.1, 22.7, 21.4, 14.2; ESI-HRMS (M + H)^+^*m/z* calcd for C_21_H_36_NO_2_ 334.2746, found 334.2799.

1-(4-(Heptyloxy)phenethyl)-4-methylpiperidine (**7**): ^1^H NMR (500 MHz, CDCl_3_) δ 7.09 (d, *J* = 8.6 Hz, 2H), 6.78 (d, *J* = 8.6 Hz, 2H), 3.87 (t, *J* = 6.6 Hz, 2H), 3.50–3.37 (m, 2H), 3.13–2.99 (m, 4H), 2.71–2.58 (m, 2H), 1.82–1.75 (m, 4H), 1.72 (dt, *J* = 14.5, 6.6 Hz, 2H), 1.62 –1.59 (m, 1H), 1.44–1.36 (m, 2H), 1.35–1.23 (m, 6H), 0.98 (d, *J* = 6.5 Hz, 3H), 0.85 (t, *J* = 6.9 Hz, 3H); ^13^C NMR (125 MHz, CDCl_3_) δ 158.3, 129.9, 127.8, 114.9, 78.1, 46.5, 33.4, 31.8, 29.3, 29.1, 26.1, 22.7, 14.1; ESI-HRMS (M + H)^+^*m/z* calcd for C_21_H_36_NO 318.2797, found 318.2712.

(1-(4-(Heptyloxy)phenethyl)piperidin-3-yl)methanol (**8**): ^1^H NMR (500 MHz, CDCl_3_) δ 7.04 (d, *J* = 8.6 Hz, 2H), 6.75 (d, *J* = 8.7 Hz, 2H), 3.86 (t, *J* = 6.6 Hz, 2H), 3.62 (dd, *J* = 11.3, 4.5 Hz, 2H), 3.48–3.41 (m, 1H), 3.39–3.29 (m, 1H), 3.08–2.96 (m, 4H), 2.69–2.54 (m, 2H), 2.33–2.21 (m, 2H), 2.01 (ddd, *J* = 26.2, 8.0, 5.5 Hz, 1H), 1.88–1.80 (m, 1H), 1.73 (dt, *J* = 14.6, 6.6 Hz, 3H), 1.41 (dq, *J* = 9.1, 6.7 Hz, 2H), 1.35–1.22 (m, 6H), 0.87 (t, *J* = 6.9 Hz, 3H); ^13^C NMR (125 MHz, CDCl_3_) δ 158.2, 129.9, 125.9, 114.9, 68.1, 64.2, 59.4, 56.0, 53.4, 31.9, 29.8, 29.4, 29.2, 26.1, 22.7, 21.4, 14.2; ESI-HRMS (M + H)^+^*m/z* calcd for C_21_H_36_NO_2_ 334.2746, found 334.2762.

1-(4-(Heptyloxy)phenethyl)piperazine (**9**): ^1^H NMR (500 MHz, CDCl_3_) δ 7.02 (d, *J* = 8.2 Hz, 2H), 6.79 (d, *J* = 8.4 Hz, 2H), 3.90 (t, *J* = 6.6 Hz, 2H), 3.32, -3.20 (m, 4H), 2.83–2.71 (m, 4H), 2.67–2.55 (m, 4H), 1.79–1.68 (m, 2H), 1.47–1.38 (m, 2H), 1.38–1.21 (m, 6H), 0.88 (t, *J* = 6.7 Hz, 3H); ^13^C NMR (125 MHz, CDCl_3_) δ 157.6, 129.2, 125.9, 114.6, 68.1, 59.9, 49.6, 43.7, 31.9, 29.4, 29.2, 26.1, 22.7, 21.4, 14.2; ESI-HRMS (M + H)^+^*m/z* calcd for C_19_H_33_N_2_O 305.2593, found 305.2587.

2-(4-(4-(Heptyloxy)phenethyl)piperazin-1-yl)ethanol (**10**): ^1^H NMR (500 MHz, CDCl_3_) δ 7.06 (d, *J* = 8.6 Hz, 2H), 6.79 (d, *J* = 8.6 Hz, 2H), 3.89 (t, *J* = 6.6 Hz, 2H), 3.72–3.65 (m, 2H), 2.79–2.59 (m, 14H), 1.78–1.69 (m, 2H), 1.46–1.36 (m, 2H), 1.36–1.21 (m, 6H), 0.86 (t, *J* = 6.9 Hz, 3H); ^13^C NMR (125 MHz, CDCl_3_) δ 157.7, 129.6, 126.0, 114.6, 68.1, 60.2, 59.4, 57.5, 52.6, 52.4, 32.3, 31.9, 29.4, 29.1, 26.1, 22.7, 14.2; ESI-HRMS (M + H)^+^*m/z* calcd for C_21_H_37_N_2_O_2_ 349.2855, found 349.2817.

(*R*)-4-(2-(2-(Hydroxymethyl)pyrrolidin-1-yl)ethyl)phenyl benzenesulfonate (**11**): ^1^H NMR (500 MHz, CDCl_3_) δ 7.84–7.76 (m, 2H), 7.68–7.61 (m, 1H), 7.55–7.47 (m, 2H), 7.11 (d, *J* = 8.5 Hz, 2H), 6.87 (d, *J* = 8.6 Hz, 2H), 3.71 (dd, *J* = 12.0, 3.1 Hz, 1H), 3.59 (dd, *J* = 12.1, 4.7 Hz, 1H), 3.48 (dd, *J* = 9.6, 4.4 Hz, 1H), 3.24–3.13 (m, 1H), 3.02 (dd, *J* = 19.1, 9.9 Hz, 2H), 2.91 (ddd, *J* = 13.6, 10.5, 5.1 Hz, 1H), 2.84–2.73 (m, 1H), 2.58 (dd, *J* = 16.8, 8.3 Hz, 1H), 2.04–1.80 (m, 4H); ^13^C NMR (125 MHz, CDCl_3_) δ 148.3, 135.4, 134.3, 130.0, 129.2, 128.5, 122.6, 67.9, 61.3, 56.8, 54.5, 33.1, 29.8, 27.0, 23.9; ESI-HRMS (M + H)^+^ *m/z* calcd for C_19_H_24_NO_4_S 362.1426, found 362.1404.

4-(2-(Piperidin-1-yl)ethyl)phenyl benzenesulfonate (**12**): ^1^H NMR (500 MHz, CDCl_3_) δ 7.84–7.78 (m, 2H), 7.69–7.62 (m, 1H), 7.50 (dd, *J* = 11.1, 4.8 Hz, 2H), 7.11 (d, *J* = 8.5 Hz, 2H), 6.87 (d, *J* = 8.5 Hz, 2H), 2.89 (dd, *J* = 10.1, 6.5 Hz, 2H), 2.67 (dd, *J* = 7.2, 3.6 Hz, 2H), 2.65–2.56 (m, 4H), 1.77–1.69 (m, 4H), 1.56–1.44 (m, 2H); ^13^C NMR (125 MHz, CDCl_3_) δ 148.1, 135.5, 134.3, 130.1, 129.2, 128.5, 122.5, 60.3, 54.3, 32.0, 25.0, 23.7; ESI-HRMS (M + H)^+^ *m/z* calcd for C_19_H_24_NO_3_S 346.1477, found 346.1439.

4-(2-(4-Hydroxypiperidin-1-yl)ethyl)phenyl benzenesulfonate (**13**): ^1^H NMR (500 MHz, CDCl_3_) δ 7.82 (dd, *J* = 8.3, 1.0 Hz, 2H), 7.69–7.62 (m, 1H), 7.52 (t, *J* = 8.0 Hz, 2H), 7.12 (d, *J* = 8.5 Hz, 2H), 6.88 (d, *J* = 8.5 Hz, 2H), 3.93–3.82 (m, 1H), 2.98 (dd, *J* = 13.4, 5.5 Hz, 2H), 2.95–2.87 (m, 2H), 2.79–2.70 (m, 2H), 2.67–2.53 (m, 2H), 2.13–2.03 (m, 2H), 1.77–1.68 (m, 2H); ^13^C NMR (125 MHz, CDCl_3_) δ 148.2, 135.5, 134.3, 130.0, 129.2, 128.7, 122.6, 65.0, 59.4, 50.1, 44.2, 32.7, 31.9; ESI-HRMS (M + H)^+^ *m/z* calcd for C_19_H_24_NO_4_S 362.1426, found 362.1492.

4-(2-Morpholinoethyl)phenyl benzenesulfonate (**14**): ^1^H NMR (500 MHz, CDCl_3_) δ 7.81 (dd, *J* = 8.5, 1.2 Hz, 2H), 7.64 (ddt, *J* = 8.7, 7.2, 1.2 Hz, 1H), 7.54–7.48 (m, 2H), 7.09 (d, *J* = 8.6 Hz, 2H), 6.87 (d, *J* = 8.6 Hz, 2H), 3.75–3.65 (m, 4H), 2.73 (dd, *J* = 9.4, 6.7 Hz, 2H), 2.54–2.50 (m, 2H), 2.47 (dd, *J* = 8.9, 4.7 Hz, 4H); ^13^C NMR (125 MHz, CDCl_3_) δ 147.9, 139.4, 135.6, 134.2, 129.9, 129.2, 128.6, 122.3, 67.0, 60.5, 53.7, 32.7; ESI-HRMS (M + H)^+^ *m/z* calcd for C_18_H_22_NO_4_S 348.1270, found 348.1217.

(*S*)-4-(2-(2-(Hydroxymethyl)pyrrolidin-1-yl)ethyl)phenyl benzenesulfonate (**15**): ^1^H NMR (500 MHz, CDCl_3_) δ 7.84–7.76 (m, 2H), 7.69–7.60 (m, 1H), 7.50 (dd, *J* = 8.4, 7.8 Hz, 2H), 7.11 (d, *J* = 8.9 Hz, 2H), 6.88 (d, *J* = 8.8 Hz, 2H), 3.72 (dd, *J* = 12.6, 3.1 Hz, 1H), 3.66–3.57 (m, 1H), 3.54–3.42 (m, 1H), 3.27–3.15 (m, 1H), 3.10–2.98 (m, 2H), 2.91 (ddd, *J* = 11.2, 8.4, 5.2 Hz, 1H), 2.82–2.73 (m, 1H), 2.63–2.52 (m, 1H), 2.04–1.79 (m, 4H); ^13^C NMR (125 MHz, CDCl_3_) δ 148.3, 135.4, 134.3, 129.9, 129.2, 128.6, 122.7, 68.0, 61.4, 56.9, 54.5, 33.1, 29.8, 27.0, 23.9; ESI-HRMS (M + H)^+^*m/z* calcd for C_19_H_24_NO_4_S 362.1426, found 362.1427.

4-(2-(4-(Hydroxymethyl)piperidin-1-yl)ethyl)phenyl benzenesulfonate (**16**): ^1^H NMR (500 MHz, CDCl_3_) δ 7.84–7.78 (m, 2H), 7.65 (ddd, *J* = 7.2, 2.4, 1.2 Hz, 1H), 7.54–7.48 (m, 2H), 7.12 (d, *J* = 8.5 Hz, 2H), 6.87 (d, *J* = 8.6 Hz, 2H), 3.49 (d, *J* = 5.3 Hz, 2H), 3.29 (d, *J* = 11.6 Hz, 2H), 2.96 (dd, *J* = 10.5, 5.7 Hz, 2H), 2.83 (dd, *J* = 11.3, 5.2 Hz, 2H), 2.41 (t, *J* = 9.8 Hz, 2H), 1.84 (d, *J* = 10.6 Hz, 2H), 1.66–1.55 (m, 3H); ^13^C NMR (125 MHz, CDCl_3_) δ 148.3, 135.4, 134.4, 130.1, 129.3, 128.5, 122.6, 66.7, 59.2, 53.2, 37.3, 31.3, 29.8, 27.2; ESI-HRMS (M + H)^+^*m/z* calcd for C_20_H_26_NO_4_S 376.1583, found 376.1523.

4-(2-(4-Methylpiperidin-1-yl)ethyl)phenyl benzenesulfonate (**17**): ^1^H NMR (500 MHz, CDCl_3_) δ 7.83–7.79 (m, 2H), 7.65 (td, *J* = 7.6, 1.1 Hz, 1H), 7.54–7.48 (m, 2H), 7.12 (d, *J* = 8.4 Hz, 2H), 6.87 (d, *J* = 8.6 Hz, 2H), 3.16 (d, *J* = 11.5 Hz, 2H), 2.94 (dd, *J* = 11.3, 5.4 Hz, 2H), 2.74 (dd, *J* = 10.1, 6.4 Hz, 2H), 2.27 (t, *J* = 11.1 Hz, 2H), 1.71 (d, *J* = 11.8 Hz, 2H), 1.52 (ddd, *J* = 25.6, 8.9, 0.9 Hz, 3H), 0.96 (d, *J* = 6.0 Hz, 3H); ^13^C NMR (125 MHz, CDCl_3_) δ 148.2, 135.5, 134.3, 130.1, 129.2, 128.6, 122.5, 59.6, 53.5, 32.7, 31.7, 30.1, 21.4; ESI-HRMS (M + H)^+^*m/z* calcd for C_20_H_26_NO_3_S 360.1633, found 360.1634.

4-(2-(3-(Hydroxymethyl)piperidin-1-yl)ethyl)phenyl benzenesulfonate (**18**): ^1^H NMR (500 MHz, CDCl_3_) δ 7.88–7.80 (m, 2H), 7.65 (ddt, *J* = 8.7, 7.2, 1.2 Hz, 1H), 7.54–7.47 (m, 2H), 7.08 (d, *J* = 8.6 Hz, 2H), 6.86 (d, *J* = 8.6 Hz, 2H), 3.62 (dd, *J* = 10.8, 5.0 Hz, 1H), 3.52–3.43 (m, 1H), 3.07 (d, *J* = 10.3 Hz, OH), 2.89–2.79 (m, 2H), 2.68–2.62 (m, 2H), 2.61–2.47 (m, 4H), 2.28 (t, *J* = 10.0 Hz, 1H), 2.21–2.11 (m, 1H), 1.96–1.86 (m, 1H), 1.83–1.75 (m, 1H), 1.73–1.64 (m, 1H); ^13^C NMR (125 MHz, CDCl_3_) δ 148.1, 138.7, 135.5, 134.3, 130.1, 129.2, 128.5, 125.9, 122.4, 66.4, 60.3, 57.1, 54.2, 37.7, 32.2, 26.9, 24.1; ESI-HRMS (M + H)^+^*m/z* calcd for C_20_H_26_NO_4_S 376.1583, found 376.1555.

4-(2-(Piperazin-1-yl)ethyl)phenyl benzenesulfonate (**19**): ^1^H NMR (500 MHz, CDCl_3_) δ 7.89–7.81 (m, 2H), 7.68–7.61 (m, 1H), 7.51 (t, *J* = 8.1 Hz, 2H), 7.05 (d, *J* = 8.9 Hz, 2H), 6.87 (d, *J* = 8.9 Hz, 2H), 3.25–3.15 (m, 4H), 2.69 (dd, *J* = 11.6, 4.4 Hz, 8H) ^13^C NMR (125 MHz, CDCl_3_) δ 148.1, 138.8, 135.6, 134.3, 130.6, 129.8, 129.3, 129.0, 125.9, 122.4, 59.4, 50.1, 44.1, 32.6; ESI-HRMS (M + H)^+^*m/z* calcd for C_18_H_23_N_2_O_3_S 347.1429, found 347.1484.

4-(2-(4-(2-Hydroxyethyl)piperazin-1-yl)ethyl)phenyl benzenesulfonate (**20**): ^1^H NMR (500 MHz, CDCl_3_) δ 7.80 (dd, *J* = 8.5, 1.2 Hz, 2H), 7.67–7.59 (m, 1H), 7.49 (dd, *J* = 8.3, 7.5 Hz, 2H), 7.07 (d, *J* = 8.6 Hz, 2H), 6.85 (d, *J* = 8.6 Hz, 2H), 3.64–3.55 (m, 2H), 2.76–2.65 (m, 4H), 2.58–2.43 (m, 10H); ^13^C NMR (125 MHz, CDCl_3_) δ 147.9, 139.5, 135.6, 134.2, 130.0, 129.2, 128.6, 122.3, 60.0, 59.4, 57.8, 53.1, 52.9, 32.9; ESI-HRMS (M + H)^+^*m/z* calcd for C_20_H_27_N_2_O_4_S 391.1692, found 391.1602.

### 2.2. Biology

#### 2.2.1. Chemicals and Reagents

DMEM, RPMI, trypsin-EDTA 0.25% and penicillin-streptomycin (PS) was purchased from GE Healthcare Life Sciences Hyclone Laboratories (Pittsburg, PA, USA). Fetal bovine serum (FBS) was received from Gibco (Thermo Fisher Scientific Inc., Walthem, MA, USA). ApoScan^TM^ annexin V-FITC was obtained from BioBud (Cat. No.: LS-02-100, Seongnam, Gyeonggi-do, Korea). MTT cell viability assay kit EZ-CYTOX was acquired from Do-GenBio Co. Ltd. (Seoul, Korea). PP2A activity kit was purchased from Millipore Corporation (Billerica, MA, USA). Antibodies against AKT (9272), pAKT (Thr308) (13038), Caspase-3 (9662), BAX (2772), and PARP (9542), and Bcl-2 (3498) were provided from Cell Signaling Technology (Danvers, MA, USA). *β*-actin (sc-47778), cytochrome-c (sc-13156), ERK 1/2 (sc-514302), pERK (Thr202/Tyr204) (sc-136521), and HRP-conjugated anti-mouse and anti-rabbit antibodies were received from Santa Cruz Biotechnology (Dallas, TX, USA). SK1 (ab71700) and SK1 (ab264042) antibodies were acquired from Abcam (Cambridge, UK). Protein marker, protease, and phosphatase inhibitor cocktail were purchased from Thermo Fisher Scientific (Waltham, MA, USA). ECL Solution for western blotting imaging was received from Millipore Corporation (Burlington, MA, USA).

#### 2.2.2. Cell Culture and MTT Cell Viability Assay

The HT29 and HCT116 cells (human colon cancer), AGS (human gastric cancer), and MIA PaCa-2 cells (human pancreatic cancer) were purchased from Korean Cell Line Bank (Seoul, Korea). Cancer cells were maintained in RPMI (HT29 and AGS) and DMEM (HCT116 and MIA PaCa-2) culture media supplemented with 10% fetal bovine serum (FBS) and 100 U/mL penicillin and streptomycin at 37 °C in a 95% humidified incubator containing 5% CO_2_. The cytotoxic effect of the compounds was assessed using the MTT cell viability assay. Briefly, 3 × 10^3^ cells/well were grown in 96-well plates for 24 h. After treatment with desired concentration of respective compounds for 24 h or 48 h, the EZ-CYTOTOX (10 µM) reagent was added in each well and incubated for 90 min. The absorbance was measured at 450 nm wavelength using Skanlt software 5.0 (Thermo Scientific, Waltham, MA, USA) installed on a Multiskan GO (Thermo Scientific, Waltham, MA, USA) spectrophotometer. Each experiment was independently repeated three times (*n* = 12).

#### 2.2.3. Clonogenic Assay

Cell proliferation was assayed by in vitro clonogenic assay. To perform the clonogenic assay, HT29 and HCT116 cells (1000 cells/well) were grown in 6-well plates for 24 h. Cells were treated with compound **10** and FTY720 in different concentrations for 12 h and changed fresh medium. Colonies were allowed to grow for two weeks by changing to a fresh medium every 3 days. Colonies were stained with 0.1% crystal violet prepared in 25% keto-glutaraldehyde for 30 min and washed with running water and photographed with Canon Rebel T6i (Tokyo, Kanto, Japan). Colonies were counted using Image J software version 1.53i, National Institutes of Health, Bethesda, MD, USA). Plating efficiency (PE) and surviving fraction (SF) was calculated using the following formula.

(1) $$\left[\mathrm{Plating}\;\mathrm{efficiency}\;\left(\mathrm{PE}\right)\right]=\frac{\mathrm{Number}\;\mathrm{of}\;\mathrm{colony}\;\mathrm{count}\;\mathrm{in}\;\mathrm{control}}{\mathrm{Number}\;\mathrm{of}\;\mathrm{cell}\;\mathrm{seeded}}$$

$$\left[\mathrm{Surviving}\;\mathrm{Fraction}\right]=\frac{\mathrm{Number}\;\mathrm{of}\;\mathrm{colony}\;\mathrm{count}\;}{\mathrm{Number}\;\mathrm{of}\;\mathrm{cell}\;\mathrm{seeded}\;\times \mathrm{PE}\;}$$

#### 2.2.4. Annexin V-FITC Staining

After treating HT29 and HCT116 cells with compounds **10**, **11**, **13** and FTY720 according to the manufacturer’s instruction, the annexin V and PI staining technique was employed to determine apoptosis in the cells. Briefly, cells (2.5 × 10^5^ cells/well) were seeded in 12-well plates and treated with 10 and/or 20 µM of compounds for 24 h. Cells were collected using trypsin-EDTA and stained with annexin V-FITC dye for 15 min at room temperature, followed by PI staining for 20 min and analyzed by flow cytometer (MACSQuant Analyzer 10; Miltenyi Biotec, Bergisch Gladbach, Germany). The data was analyzed using MACSQuantify^TM^ version 2.8 software (Miltenyi Biotec, Bergisch Gladbach, Germany) and presented as early apoptosis, late apoptosis, or combined apoptosis percentage. Each experiment was repeated three times independently (*n* = 6).

#### 2.2.5. Mitochondrial Outer Membrane Potential (MOMP) Determination by JC-10 Staining

To access the mechanism of cell death, mitochondrial outer membrane potential (MOMP) was measured using JC-10 cationic dye. HT29 and HCT116 cells (2.5 × 10^5^ cells/well) were seeded in 12-well plates and treated with a test compound for 24 h, collected in 1.5 mL microtubes using trypsin-EDTA, washed with 1× cold PBS, and stained with JC-10 dye prepared in HBSS solution (0.14 M NaCl, 0.005 M KCl, 0.001 M CaCl_2_, 0.005 M MgSO_4_. 7H_2_O, 0.0004 M MgSO_4_-6H_2_O, 0.0003 M Na_2_HPO_4_-2H_2_O, 0.0004 M KH_2_PO_4_, 0.006 M d-glucose, 0.004 M NaHCO_3_, 0.002 M HEPES, and 0.02% Pluronic^®^ F-127) for 30 min. Samples were analyzed using MACSQuanta Analyzer 10. Cells with green and red fluorescence were plotted in a dot plot; the ratio of red to green fluorescence was calculated using MACSQuantify^TM^ version 2.8 software and presented as MOMP of the cells. The experiment was repeated three times (*n* = 6).

#### 2.2.6. Western Blotting

The cell lysate was prepared after successive treatment using mammalian protein extraction buffer (Organic buffer + 10 mM NaCl + detergent pH 7.5) mixed with 1% protease/phosphatase inhibitor cocktail; protein was quantified with BCA assay, boiled at 95 °C for 10 min by adding sample loading buffer, separated using SDS-PAGE, and transferred into methanol-activated PVDF membrane. Primary antibodies were probed overnight in PVDF membrane at 4 °C and blocked with subsequent HRP anti-mouse and anti-rabbit secondary antibodies at room temperature. Then, membranes were washed with TBST buffer, and images were detected by Amersham Imager 680 (GE Healthcare; Leiden, WZ, The Netherlands) using ECL solution. Band intensities were analyzed and quantified using Image J software (version 1.53i).

#### 2.2.7. SK Activity Assay

The effect of compounds in sphingosine kinase activity was examined using the AdapdaTM screening system (Thermo Fisher Scientific System, Waltham, MA, USA). The kinase inhibition ability of compounds **1**–**11** along with RB005 was measured with final concentration of 20 μM. The SK1 enzyme activity assay was performed using 0.04–0.16 ng SK1 enzyme, 50 μM of sphingosine lipid substrate in reaction buffer containing HEPES 32.5 mM (pH 7.5), BRIJ 35 0.005%, MgCl_2_ 5 μM, and 0.5 mM ethylene glycol-bis (*β*-aminoethyl ether)-*N*,*N*,*N*′,*N*′-tetraacetic acid (EGTA), whereas 35–140 ng SK2 enzyme along with 50 μM sphingosine lipid substrate was used in 32.5 μM HEPES pH 7.5, 0.5 μM EGTA, 1.5 μM MgCl_2_ buffer to detect SK2 enzyme activity. The data were presented as percentage inhibition of SK enzyme by individual compounds.

#### 2.2.8. S1P, Sphingosine and Ceramide ELISA

The cellular levels of S1P, sphingosine, and ceramide were detected using an ELISA assay kit (MyBioSource, Inc., San Diego, CA, USA) according to the manufacturer’s instruction. For S1P ELISA, 100 µL cell lysates or standards were added in pre-coated ELISA wells, incubated for 90 min at 37 °C, and treated with 1x biotinylated anti S1P antibody for 1 h. Following treatment of HRP conjugated secondary antibody, the substrate was added and allowed to change the color for 15 min; then, the stop solution was added and absorbance was measured at 450 nm using a Multiskan GO (Thermo Scientific, Waltham, MA, USA) microplate spectrophotometer. For sphingosine, 50 µL cell lysate or standards were added to the wells, a 50 µL detection working solution was added, it was incubated for 1 h at 37 °C, a 100 µL reagent B working solution was added, and it was incubated at 37 °C for 45 min. After, a substrate solution was added and incubated at 37 °C for 20 min in the dark. A stop solution (50 µL) was added to each well, and optical density was measured at 450 nm using a microplate reader. For total ceramide ELISA assay, 100 µL sample and standards were added in appropriate wells, and a 10 µL balance solution was added in sample wells only. After, a 50 µL conjugate solution was added in each well (except blank) plate and was gently mixed and incubated at 37 °C for 1 h. After incubation, 50 µL substrate A and B was added to the plate and incubated at 37 °C for 20 min; a stop solution (50 µL) was added to each well and optical density was determined at 450 nm using a microplate reader. The concentration of S1P, sphingosine, and ceramide were calculated as ng/mL using respective standard curves constructed by Skanlt microplate reader software 5.0 (Thermo Scientific, Waltham, MA, USA).

#### 2.2.9. PP2A Assay

The activity of PP2A was measured by using the PP2A kit according to the manufacturer’s instruction. The cell lysate was incubated with anti PP2A, C subunit antibody, and protein A agarose slurry for 2 h at 4 °C. The phosphopeptide solution (60 µL) was mixed and incubated at 30 °C for 10 min in a shaker. After a brief centrifuge, a 25 µL sample was liquated in ½ volume microplate and 100 µL malachite green phosphate detection working solution was added. After color development, absorbance was taken at 650 nm in a Multiskan GO microplate reader. A phosphate standard curve was constructed using phosphate standard, and results were calculated using this standard curve. This experiment was repeated three times (*n* = 6).

#### 2.2.10. Inhibition of CYP Enzymes Activity by FTY720, PF-543, and Compound **10**

Human liver microsomes (0.25 mg/mL) and 0.1 M phosphate buffer (pH 7.4), substrate drug cocktail of 5 drug metabolites (phenacetin 50 μM, diclofenac 10 μM, S-mephenytoin 100 μM, dextromethorphan 5 μM, midazolam 2.5 μM), and FTY720 were added at concentrations of 0 and 10 μM, respectively, and incubated at 37 °C for 5 min in advance, and then NADPH generation system solution was added and incubated at 37 °C for 15 min. Afterward, to terminate the reaction, an acetonitrile solution containing an internal standard (terfenadine) was added, centrifuged for 5 min (14,000 rpm, 4 °C), and the supernatant was injected into the LC-MS/MS system. By simultaneously analyzing metabolites, the ability of the six compounds to inhibit drug metabolism enzymes was evaluated.

Metabolites of each CYP coenzyme indicator drug generated through the above reaction were analyzed using the Shimadzu Nexera XR system and TSQ vantage (Thermo). The HPLC column was a Kinetex C18 column (2.1 × 100 mm^2^, 2.6 μm particle size; Phenomenex, Torrance, CA, USA), and the mobile phase was distilled water (A) containing 0.1% formic acid and acetonitrile (B) containing 0.1% formic acid. The same gradient program was used (Table 1). The generated metabolites were quantified using MRM (Multiple Reaction Monitoring) quantification modes, and data were analyzed using Xcalibur (version 1.6.1).

#### 2.2.11. In Vitro Metabolic Stability of Compound **10**, FTY720 and PF-543

The metabolic stability of compounds **10**, PF543 and FTY720 were measured using human, mice and rat liver microsomes. Microsome incubation was measured in triplicate in a 1.5 mL Eppendorf tube with 0.1 M potassium phosphate buffer (pH 7.4). Reduced nicotinamide adenine dinucleotide phosphate (NADPH)-dependent metabolism was evaluated by incubation of FTY720, PF-543, compound **10,** or Verapamil (positive control) with pooled liver microsomes of human (HLM), rat (RLM) and mouse (MLM). It has a final volume of 100 mL in the presence of an NADPH regeneration system. In the final incubation mixture, 0.5 mg/mL HLM, 0.1 M potassium phosphate buffer (pH 7.4), and NADPH regeneration system (1 mM NADPH, 10 mM MgCl_2_) were included. In the pre-incubation mixture, HLM was added, and the mixture was run in a shaking incubator at approximately 350 rpm and 37 °C for 5 min under a Thermomixer (Eppendorf, Hamburg, Germany). Reactions were initiated by the addition of 1 mM NADPH and then quenched by the addition of 40 μL of ice-cold acetonitrile containing 10 μM chlorpropamide (CPP) as an internal standard at 0 and 30 min. After quenching, centrifugation of the incubation mixture was performed at 15,000× *g* for 5 min at 4 °C. An aliquot of 2 µL of the supernatant was injected into the LC-MS/MS system. The LC-MS/MS system was Xcalibur version 1.1.1 (Thermo Fisher Scientific Inc., Waltham, MA, USA).

#### 2.2.12. Statistical Analysis

The statistical software GraphPad Prism (v.7.0, La Jolla, CA, USA) was used to analyze the data. Unless otherwise stated, all the results were reported as means ± SD. The data with heterogeneous variance were subjected to a non–parametric one–way ANOVA. The intergroup variation was determined using Tukey’s post-procedure analysis. If *p* < 0.05, the difference was considered significant.

## 3. Results

### 3.1. Chemistry

**Scheme 1:**
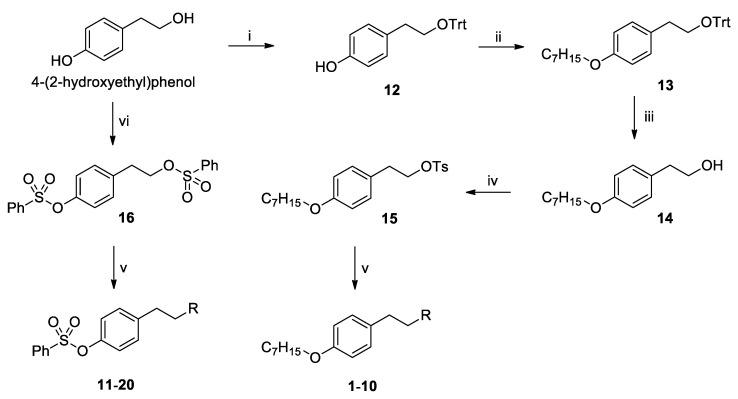
Synthesis of RB005 and PF-543 derivatives **1**–**20**. Reagents and conditions: (i) TrtCl, DMAP, CH_2_Cl_2_/pyridine (1/2), rt, 1 d, (ii) 1-bromoheptane, K_2_CO_3_, DMF, 80 °C, 12 h, (iii) conc. HCl, THF, 50 °C, 3 d, (iv) TsCl, NEt_3_, CH_2_Cl_2_, rt, 12 h, (v) cyclic amines, MeCN, 40 °C, 12 h, (vi) PhSO_2_Cl, NEt_3_, CH_2_Cl_2_, rt, 12 h.

In the synthesis method, 4-(2-hydroxyethyl) phenol was used as a starting material to introduce a triphenylmethyl group, which is a bulky protecting group to synthesize compound **12**, and a heptyl group was introduced at the phenol portion using potassium carbonate as a base. The protecting group of compound **13** was removed using HCl, and then tosylation was used to synthesize compound **14**. Finally, compounds **1**–**10** were synthesized by introducing various cyclic amines in compound **15**. Compound **16**, in which a benzenesulfonyl group was introduced into two hydroxyl groups with a yield of 89%, was synthesized by using three equivalents of benzenesulfonyl chloride at the same starting material to introduce a tail group similar to PF-543. Using compound **16**, cyclic amines were introduced in the same manner as for compounds **1**–**10** to obtain compounds **11**–**20** by a simple synthesis method (Scheme 1, Table 2).

### 3.2. Biological Evaluations

#### 3.2.1. Cytotoxic Effects of Compounds **1**–**10** in Human Colon Cancer Cells

**Figure 2:**
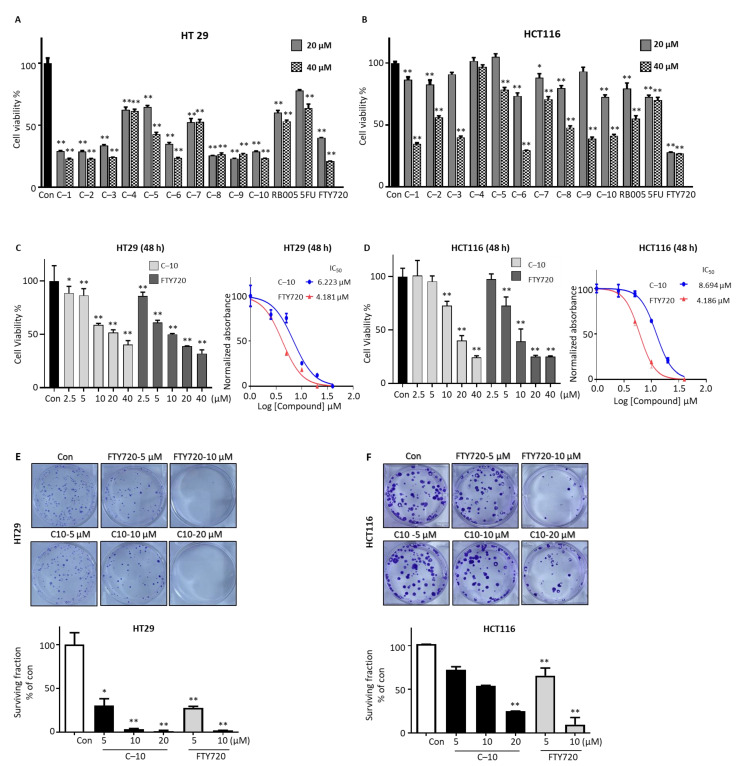
Compound **10** inhibits the growth and proliferation of colorectal cancer cells in vitro. The cell viability of (**A**) HT29 and (**B**) HCT116 with treatment of 20 and 40 µM concentration of compounds **1**–**10** for 24 h was determined by MTT cell viability assay and compared with RB005, 5-FU, and FTY720. Compound **10** was selected, concentration-dependent (2.5–40 µM) cell toxicity effect after 48 h treatment was performed, and IC_50_ was determined in (**C**) HT29 and (**D**) HCT116. Cell proliferation assay was performed after treatment of compound **10** (5 and 10 µM) and FTY720 (5 and 10 µM) in (**E**) HT29 and (**F**) HCT116 cells, and the surviving fraction was calculated as described in the method. The results presented are mean ± SD for three individual experiments. * *p* < 0.05, ** *p* < 0.01 compared with nontreated control group.

First, the synthesized compounds were separated into two sets according to their structural properties: compounds **1**–**10**, which are analogs of RB005 (FTY720 analog), and compounds **11**–**20**, which are analogs of PF-543. The cytotoxic effect of compounds **1**–**10** was observed in HT29 and HCT116 colon cancer cell lines. The cytotoxic effect of these newly synthesized compounds was compared with its structural analogs FTY720 and RB005 and widely used anti-colorectal cancer drug 5-Fluorouracil (5-FU). The initial screening of the cell viability experiment was conducted in colon cancer cell lines at concentrations of 20 and 40 μM. The overall cytotoxic effect of all compounds **1**–**10** in HT29 cells was significantly higher than in the nontreated control group (Figure 2A). Compounds **1**, **2**, **3**, **6**, **8**, **9** and **10** were more cytotoxic than RB005 and 5-FU and almost similar to the effect of FTY720 in HT29 cells. Similarly, in HCT116 cells, compounds **1**, **2**, **6**, **7**, **8** and **10** have significant cytotoxic effects in both 20 and 40 µM concentrations, and only 40 µM concentration of compounds **3**, **5** and **9** shows significant cytotoxic effect (Figure 2B).

Combining the results of all cells, we selected compound **10** for further experiments in colon cancer cells. Initially, the concentration-dependent cytotoxic effect of compound **10** was accessed in both HT29 and HCT116 cell lines. The bivariate correlation analysis confirmed the negative relation between compound **10** concentration and cell survival in both cancer cell lines (Figure 2C,D, left). The relative IC_50_ of compound **10** was measured 6.223 μM and 8.694 μM at 48 h treatment and 10.54 μM and 10.88 μM at 24 h in HT29 and HCT116 cells, respectively (Figure 2C,D, right panel and Supplementary Material Figure S1A,B). Furthermore, we performed colony formation assay after treatment of 5, 10, and 20 μM of compound **10,** and 5 and10 μM of FTY720 in HT29 and HCT116 cells for 24 h. Compound **10** effectively inhibits the colony formation capacity of HT29 and HCT116 cell lines with 5, 10 and 20 μM concentration in a dose-dependent manner, which is similar to the effect of 5 and 10 μM concentrations of FTY720 (Figure 2E,F). These results show that compound **10** effectively inhibits colon cancer cells viability and proliferation in vitro, and this inhibitory effect is dose dependent.

#### 3.2.2. Compound **10** Induced Apoptosis of Colorectal Cancer Cells through the Intrinsic Mitochondrial Pathway

**Figure 3:**
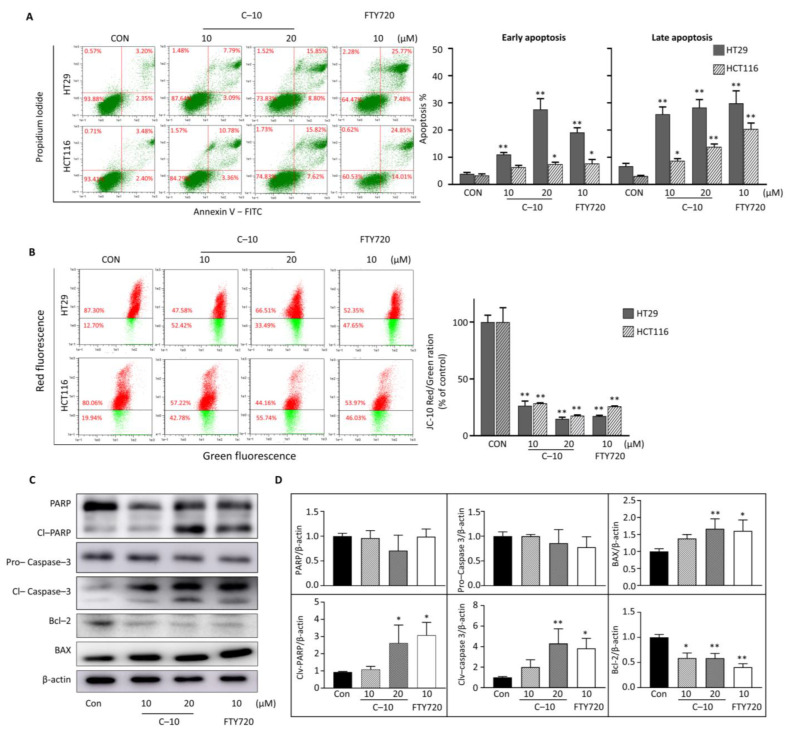
Compound **10** inhibits colorectal cancer through the intrinsic mitochondrial apoptotic pathway. (**A**) The HT29 and HCT116 cells were treated with the desired concentration of compound **10** and FTY720; cell apoptosis assay was performed by annexin–V assay and presented as early (left) and late (right) apoptosis. (**B**) Mitochondrial outer membrane potential was determined using JC−10 dye; both the samples were analyzed by flow cytometer. (**C**) HCT116 cells were treated with compound **10** and FTY720; the protein was extracted, and expression of proteins PARP, cleaved PARP, pro−caspase−3, cleaved caspase−3, Bcl−2, and BAX were determined by western blotting. *β*−actin was used as the loading control. (**D**) All the western blotting bands were quantified using Image–J software and expressed as protein/β–actin compared to the nontreated control group. The result is representative of three independent experiments, and data are presented as mean ± SD. * *p* < 0.05, ** *p* < 0.01 compared with nontreated control group.

Since the cell viability assay has confirmed that compound **10** could dose-dependently inhibit the growth of colorectal cancer cells, an annexin V assay was conducted to further identify the cell death type in colon cancer cells. Treatment of compound **10** induced apoptosis of both HT29 and HCT116 cells in a dose-dependent manner (Figure 3A). These results revealed that compound **10** follows the apoptotic pathway for cell death. As apoptosis has various signaling mechanisms, we additionally performed JC-10 staining of HT29 and HCT116 cells after treatment of compound **10** and FTY720 to determine mitochondrial outer membrane potential (MOMP). The treatment of 10 and 20 µM of compound **10** in HT29 cells and HCT116 cells significantly reduced JC-10 dye red to green fluorescence ratio compared to nontreated control cells (Figure 3B). These results show that compound **10** reduced the MOMP, increased the mitochondrial membrane’s permeability, and induced apoptosis in colorectal cancer cells. To validate the intrinsic apoptosis mechanism followed by compound **10**, we analyzed the expression of apoptotic pathway-related proteins (Figure 3C). The execution of apoptosis was demonstrated with notably decreased whole PARP protein level and increased cleaved PARP level in the compound **10**-treated group compared to nontreated groups. Treatment of 10 and 20 µM of the compound effectively increased the cleavage of apoptosis executer protein caspase-3 compared to nontreated control cells while pro-caspase protein level was reduced and increased the expression of proapoptotic protein BAX, whereas it decreased the expression of antiapoptotic Bcl-2 protein. These results show that compound **10** colorectal cancer inhibition follows the mitochondrial-mediated intrinsic apoptotic cell death pathway.

#### 3.2.3. Cytotoxic Effects of Compounds **11**–**20** in Colorectal Cancer Cells

**Figure 4:**
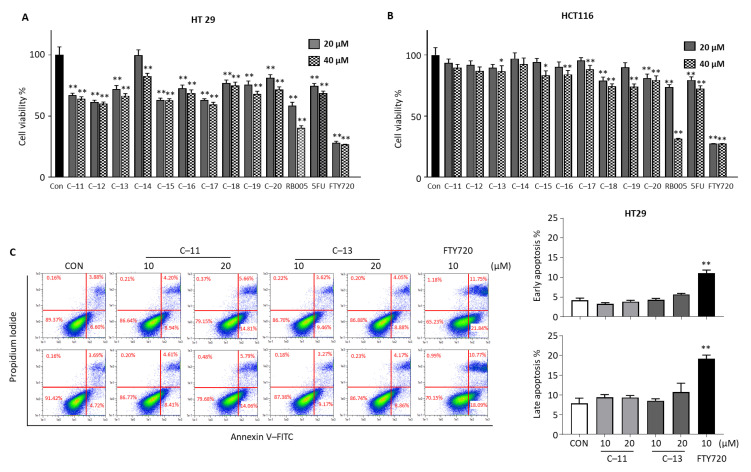
Effect of compounds **11**–**20** in colorectal cancer cells. Cytotoxicity effect of compounds **11**–**20** at 10 and 20 µM concentration for 24 h in (**A**) HT29 and (**B**) HCT116 cells were determined. (**C**) HT29 cell apoptosis effect was analyzed by annexin V assay after treatment of compounds **11** and **13** (10 and 20 µM) and FTY720 (10 µM) using flow cytometry. The result is representative of three independent experiments, and data are presented as mean ± SD. * *p* < 0.05, ** *p* < 0.01 compared with nontreated control group.

We determined the cytotoxic effect of the novel synthetic compounds **11**–**20** with benzene sulfonyl tail structure in HT29 and HCT116. In HT29 cells, most compounds **11**–**20** showed a cytotoxic effect similar to that of 5-FU (Figure 4A), whereas, in HCT116 cells, the new compounds showed lower cytotoxic effects than 5-FU (Figure 4B), so the new compounds did not appear to have a marked cytotoxic effect in colon cancer cells. To confirm the cell death mechanism in HT29 cells, we chose two compounds, **11** and **13**, then performed annexin V apoptosis assay in HT29 cells (Figure 4C). Treatment of both compounds **11** and **13** (10 and 20 µM) for 24 h did not significantly induce apoptosis in HT29 cells, whereas FTY720 (10 µM) increased apoptosis. This shows the compounds **11**–**20** with benzene sulfonyl tail structure are ineffective in colorectal cancer. To examine if they exhibit anticancer activity in additional human cancer cells, we further tested the cytotoxic effects of these compounds in human pancreatic cancer MIA PaCa-2 cells and human gastric cancer AGS cells. The overall results show that compounds **1**–**10** were more effective than compounds **11**–**20** in reducing the viability of both MIA PaCa-2 and AGS cells (Supplementary Figure S2).

#### 3.2.4. SK 1/2 Assay of Compounds

**Figure 5:**
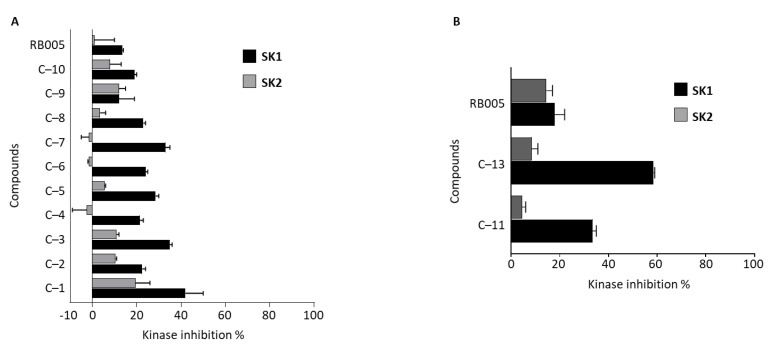
Effects of FTY720 and RB005 derivatives (20 μM) on in vitro SK1/2 activity assay. Effect of (**A**) RB005 and derivatives **1**–**10** and (**B**) PF-543 derivatives **11** and **13** on SK1/2 activity.

We measured the SK1/2 inhibitory effect of the novel synthetic compounds **1**–**10** having an aliphatic chain (Figure 5A). Compared to the RB005, which is not connected by a heteroatom, the newly synthesized compounds **1**–**10** increased the overall SK1/2 inhibitory effect. Compound **1**, which has the same head group as PF-543, showed the highest SK1 inhibitory effect, and all compounds showed selectivity to SK1. In addition, the selectivity of SK1 of all compounds was increased compared to RB005. There was no difference in the SK1/2 inhibitory effect between the compounds with hydroxy in the head group (compounds **1**, **3**, **5**, **6**, **8** and **10**) and the compounds without the hydroxy head group (compounds **2**, **4**, **7** and **9**). We measured the SK1/2 inhibitory effects of compound **11** having a head group such as PF-543 and compound **13** having a head group such as RB005 at 20 μM (Figure 5B). Compounds **11** and **13** showed a similar SK1 inhibitory effect and more selectivity than RB005. In particular, compound **11** of the head group, such as PF-543, which has the highest known SK1 inhibitory effect, showed a three-fold higher SK1 inhibitory effect than RB005.

#### 3.2.5. Compound **10** Inhibits Intracellular S1P Production and Increases Proapoptotic Sphingolipids

**Figure 6:**
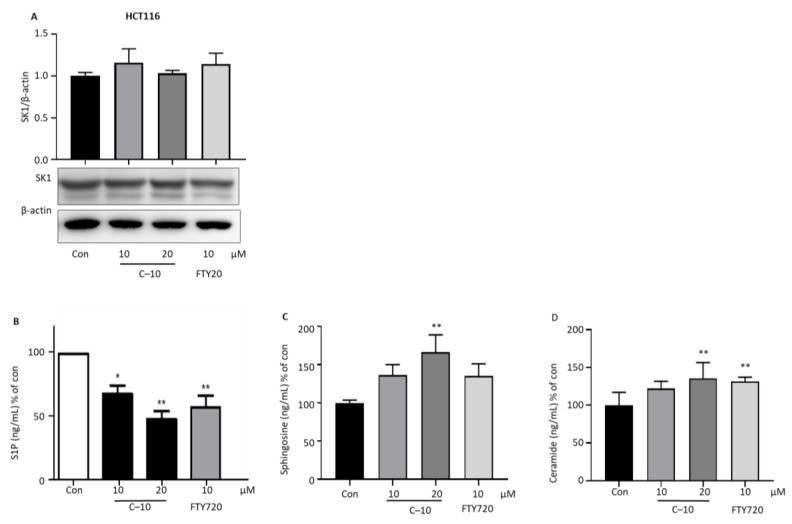
Effect of compound **10** on SK1 expression and sphingolipid-related molecules. HCT116 cells were seeded in 12-well plates and treated with compound **10** (10 and 20 µM) and FTY720 (10 µM) for 24 h. (**A**) Protein was extracted and performed western blotting of SK1 and quantified as SK1/*β*-actin compared to nontreated control cells. *β*-actin was used as the loading control. ELISA was performed for the detection of (**B**) S1P, (**C**) sphingosine and (**D**) ceramide level. The result is representative of three independent experiments, and data are presented as mean ± SD. * *p* < 0.05, ** *p* < 0.01 compared with nontreated control group.

We observed the protein expression levels of SK1 and 2 to determine whether compound **10** could reduce SK activity by reducing the expression of SK1 and 2. The treatment of 10 and 20 µM of compound **10** in HCT116 cells does not affect the SK1 protein expression level compared to control cells, which is similar to the FTY720-treated cells (Figure 6A). Treatment of compound **10** at 10 and 20 µM significantly reduces the S1P levels produced by SK in a concentration-dependent manner compared to nontreated control cells (Figure 6B). In reverse, 10 µM and 20 µM of compound **10** treatment in HCT116 cells increased sphingosine level by 1.42 and 1.74 fold compared to nontreated control cells (Figure 6C). Ceramide level was increased in HCT116 treated with 20 µM of compound **10** (Figure 6D). These results indicate that the SK1 protein expression is not affected by the treatment of compound **10.** In contrast, the apoptosis in colon cancer cells by compound **10** could be initiated due to the inhibition of SK1 enzyme activity ensuing a decreased pro-survival S1P lipid mediator level and increased proapoptotic sphingosine and ceramide level within colon cancer cells.

#### 3.2.6. PP2A Activation Is an Additional Pathway Followed by Compound **10** for Advanced Anticancer Effect

**Figure 7:**
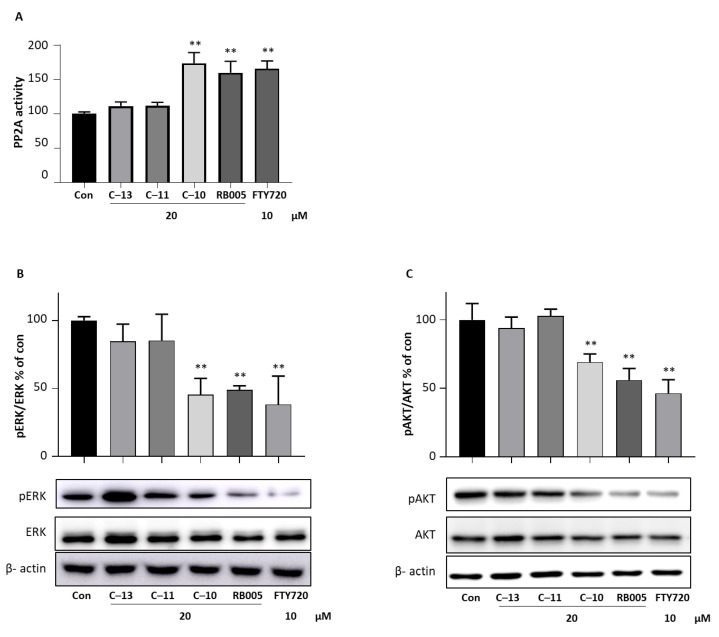
Effect of compound **10** on PP2A activity. (**A**) HCT116 cells were seeded in 12-well plates and treated with 20 µM of compounds **10**, **11,** and **13** for 24 h. Cell lysate was performed PP2A activity assay by immunoprecipitation PP2A activity assay kit. HCT116 cells were treated with 20 µM of compounds **10**, **11**, **13,** and RB005 and 10 µM of FTY720. Expression of (**B**) ERK and pERK and (**C**) AKT and pAKT was determined and quantified. *β*-actin was used as the loading control. The result is representative of three independent experiments, and data are presented as mean ± SD. ** p < 0.01 compared with nontreated control group.

The cell cytotoxic effects of the novel synthetic compounds **10**, **11**, **13**, and known SK1 inhibitors RB005 and PF-543 were compared at 10, 20 and 40 µM concentrations. The cytotoxic effects of compounds **10** and RB005 with an aliphatic tail structure were higher than compounds **11**, **13**, and PF-543 with a benzene sulfonyl tail structure in all concentration ranges (Supplementary Figure S1C). Previously, we have shown that RB005 has a higher anticancer effect in colon cancer than PF-543, and PP2A activation was the alternative pathway followed by RB005 but not by PF-543 [13]. Similarly, we conducted a PP2A activity assay of compounds **10**, **11** and **13** along with RB005 and FTY720 as a positive control in HCT116 cells. Treatment of compounds **11** and **13** did not increase PP2A activity in HCT116 cells, while compound **10** significantly increased PP2A activity by 1.73 fold compared to nontreated control cells (Figure 7A). RB005 and FTY720 increased PP2A activity by 1.59 and 1.65 fold, respectively, compared to control cells. To further validate the activity against PP2A, we observed the phosphorylation levels of AKT (Thr508) and ERK (Thr202), which PP2A dephosphorylates. Treatment of compound **11** and **13** did not decrease phosphorylation of AKT and ERK in HCT116 cells, whereas compound **10** treatments significantly reduced phosphorylation of AKT and ERK, similar to RB005 and FTY720 (Figure 7B,C). In sum, compounds (RB005 and compound **10**) containing an aliphatic chain structure show relatively higher PP2A activity than PF-543-type compounds, supporting better anticancer effect of compound **10** compared with compounds **11** and **13**.

#### 3.2.7. Inhibition of CYP Coenzyme Activity and Metabolic Stability by Compound **10**, FTY720, and PF-543

The inhibitory ability of each CYP coenzyme activity by compound **10**, FTY720, and PF-543 was expressed as percentage activity against the control without the addition of inhibitor. Compound **10** and FTY720 showed no inhibitory activity against CYP coenzyme or merely weak inhibitory activity. PF-543 exhibits an inhibitory activity of 50% or more (IC_50_ < 10 μM) against CYP2C9, 2C19 and 3A4 (Table 3). For the accuracy of our experiment, ketoconazole, a well-known existing CYP3A4 inhibitor, was added as a positive control. As a result, similar research results were obtained. 

The metabolic stability of compound **10**, FTY720, and PF-543 was expressed as % activity against the unreacted control. In vitro profile of FTY720, PF-543, and compound **10** was determined using human (HLM), rat (RLM) and mouse (MLM) liver microsomal stability (% remaining after 30 min). Compound **10** was stable in human species but unstable in rats and mice liver microsomal stability. FTY720, which is already used as a drug, was observed to be stable in the three evaluated species. The liver microsomal stability of PF-543was unstable in all species (Table 4).

## 4. Discussion

In this study, RB005 derivatives (compounds **1**–**10**) and PF-543 derivatives (compounds **11**–**20**), well-known as SK1 inhibitors, were synthesized using the same starting material, and the efficacy of these compounds on the cell cytotoxic effect of colorectal cancer cells was verified. Among **1**–**10**, compound **10** showed the best cytotoxic effect in HT29 and HCT116 cells after 24 h and 48 h. To endorse whether cytotoxicity exhibited by compound **10** was due to apoptosis, annexin V-FITC assay and apoptotic signaling molecule expression were determined. Compound **10** is a derivative of RB005, but RB005 is also an analog of FTY720, and FTY720 is superior to RB005 in cytotoxic effect, so the efficacy of apoptosis was verified compared with FTY720. It showed a similar apoptotic effect with FTY720, and an increase in the proapoptotic protein BAX and PARP cleavage by caspase-3 was also observed. Using the same starting material as the synthesis method for compounds **1**–**10**, analogs **11**–**20** with benzenesulfonyl tail similar to PF-543 were synthesized. Most of the PF-543 analogs showed lower anticancer activity than FTY720 or RB005, which have aliphatic chains, and lower cell cytotoxic effect than 5-Fluorouracil. As a result of the apoptosis efficacy, it was found that the cytotoxic effects of compounds having an aliphatic tail, such as FTY720 and RB005, were better. Therefore, to increase the apoptosis efficacy, maintaining the aliphatic structure can be expected to have an improvement effect through a synthetic approach. We conducted the SK1/2 inhibitory effect of the first set of synthesized compounds together with the known SK1 inhibitor RB005. Most of the RB005 derivatives showed improved SK1 inhibitory ability than RB005. However, it was found that the SK1 inhibitory ability and the cytotoxic effect were not directly proportional. SK inhibitors inhibit the transformation of sphingosine into S1P in cancer cells and show anticancer activity, as S1P is a well-known lipid modulator involved in cancer cell survival and proliferation [14]. Since compound **10** has an SK inhibitory effect, it can be observed that the level of S1P is decreased and that the level of sphingosine and ceramide, which are apoptosis factors, is increased. PF-543 analogs had a more SK1 selectivity and SK1 inhibitory effect than RB005. In particular, compared with compound **10**, it can be seen that compounds **11** and **13**, which are two types of PF-543 analogs, improved the inhibitory effect and selectivity of SK1. However, in terms of cytotoxicity and apoptosis, it was found that compound **10** was superior to the PF-543 analogs. These results are consistent with a previous report [14] of low anticancer activity compared to the excellent SK1 inhibitory effect of PF-543. As a result, it can be suggested that the introduction of the benzenesulfonyl tail is helpful for SK1 selectivity and activity, but the introduction of the aliphatic tail structure is more important to improve the in vitro apoptosis efficacy.

Previously, we have developed numerous SK inhibitors and tested them in various cancer cells [7,15,16,17,18]. These experiences provide us with much information about the role of sphingosine kinase inhibitor’s structure and its anticancer properties. PF-543 is a selective and most potent SK1 inhibitor; however, its anticancer response was lower than that of FTY720 and RB005, which are less potent SK1 inhibitors. Our results revealed that compound **10** had a stronger cytotoxic effect than SK1 inhibitory ability. The discrepancy between these results suggests that a mechanism other than SK inhibitory efficacy is involved in the cancer cell cytotoxic efficacy of SK inhibitors. In a previous study, we showed that PP2A activation of FTY720 and RB005 is an additional compensatory mechanism for their superior anticancer properties in colorectal cancer [13].

PP2A acts as a tumor suppressor by regulating many signaling pathways, and a decrease in PP2A function affects cancer cell transformation [19]. The correlation between the activity of PP2A and cancer has been reported in several cancers [20]. There is some evidence suggesting a relevant role of the PP2A deregulation in colorectal cancer pathogenesis, and the increase in PP2A activity by FTY720 inhibits cell growth in colorectal cancer cells. Therefore, activation of PP2A can be a drug target for colorectal cancer treatment. An experiment was conducted to confirm whether compound **10**, an analog of RB005 and FTY720, and compound **11,** with a tail structure of PF-543, activate PP2A like FTY720 to show anticancer activity. As a result, it was found that PP2A was activated in compound **10** treated with HCT116 cell lines. Because of treatment with compound **10** in the HCT116 cell line, PP2A activity was observed similar to RB005 and FTY720. Activation of PP2A by compound **10** increases the dephosphorylation of ERK and AKT, which are factors that promote cancer cell growth, thereby inducing apoptosis and contributing to inhibit cell growth. The results of PP2A activation explain the enhancement of anticancer activity in colorectal cancer of synthetic compounds. Therefore, the activity of PP2A may be more effective in anticancer activity than the inhibition of SK1. In addition, it is possible that the aliphatic chain of the FTY720 form is structurally effective in obtaining the result of PP2A activation. To reliably prove these results, more results on the synthesis of derivatives are needed.

For a compound to be developed as a drug, improving pharmacokinetics is one of the important issues. The enzymatic protein superfamily cytochrome P450 (CYP) are involved in the metabolism of drugs accompanying variability of drug pharmacokinetics and response. The major forms of CYP enzyme expressed in the liver are CYP1A2, CYP2C9, CYP2C19, CYP2D6 and CYP3A4; they are involved in drug metabolism and determine the fate of the drug adverse reactions or therapeutic failure [21,22]. Since compound **10** did not affect the activity of major CYP enzymes, consequently, it is judged that there is slight possibility of drug interaction with drugs metabolized by these coenzymes. In general, the percentage of control activity or IC_50_ value is affected by the concentration of the substrate drug. To predict in vitro to in vivo interactions, an additional study obtaining the Ki (inhibition constant) value (full enzyme inhibition study) may be needed. In addition, to predict the possibility of drug interaction more accurately, it is more important to know the concentration of test compounds in plasma and in the liver tissue, so further studies are considered necessary.

Metabolic stability is a factor that can affect PK parameters, such as drug clearance, half-life, and oral bioavailability, and is considered an important characteristic of drug candidates for defining pharmacological and toxicological profiles as well as patient compliance of a drug [23]. This experiment predicted the degree of drug metabolism by the liver, a major organ involved in drug metabolism, through an in vitro experiment. Metabolically active systems, such as liver microsomes or hepatocytes, are used to evaluate the metabolic stability of drugs. To avoid the problem of metabolic potential, a compound with a value of at least 60% for 30 min is ideal. Still, liver microsomal stability may vary depending on the conditions of each compound. Compound **10** showed CYP enzymatic activity similar to that shown by FTY720, which has already been developed as a drug, and showed excellent microsomal stability in human (HLM) compared with the known SK1 inhibitor PF-543. These results suggest that the tail structure having a heteroatom is a relatively stable drug, but in vivo PK studies are needed to reflect PK information for drug designing and synthesis.

## 5. Conclusions

The newly synthesized compounds **1**–**10** showed an overall increase in SK1/2 inhibitory effect compared to RB005 in the aliphatic tail, and, in particular, compound **3** in the head group such as RB005 showed three times higher SK1/2 inhibitory effect than RB005. These results show that the compounds of the tail structure connected by the heteroatom influence the increase of the SK1/2 inhibitory effect as well as the synthetic convenience. In addition, there was no difference in the SK1/2 inhibitory effect according to the head group, which shows that the change in the tail group is more effective in the SK1/2 inhibitory effect.

Compound **11** with a tail and head structure like PF-543 demonstrated higher SK1 selectivity than SK2 but showed low anticancer activity results. On the other hand, compound **10** with an aliphatic chain showed relatively high PP2A activity. This shows that the introduction of aliphatic chains is structurally effective in developing anticancer drugs using SK1 inhibitors.

In this study, we showed that compound **10**, a novel SK1 inhibitor with aliphatic chains linked by a heteroatom, improved anticancer activity compared to RB005. In addition, compound **10** showed metabolic stability similar to that of FTY720 in liver microsomes of humans and low inhibitory ability to CYP enzymes. These results could be used as important information for designing new anticancer active substances using SK1 inhibitory effect and PP2A activation.

## Figures and Tables

**Table 1:** The HPLC gradient solvent program.

Time (min)	Flow (mL/min)	% A	% B
0	0.3	100	0
1.0	0.3	60	40
4.0	0.3	50	50
4.1	0.3	100	0
7.0	0.3	100	0

**Table 2:** Synthesized compounds **1**–**20**.

Head Group (R)	Compound No.	Compound No.
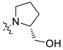	**1**	**11**
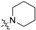	**2**	**12**
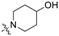	**3**	**13**
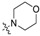	**4**	**14**
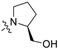	**5**	**15**
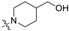	**6**	**16**
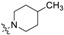	**7**	**17**
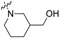	**8**	**18**
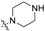	**9**	**19**
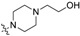	**10**	**20**

**Table 3:** Effect of compound **10**, FTY720, and PF-543 in CYP enzyme activity (% of control activity).

	CYP1A2	CYP2C9	CYP2C19	CYP2D6	CYP3A4
Compound **10**	>100	>100	96.5	87.4	>100
FTY720	86.5	96.8	86.8	92.0	>100
PF543	80.9	9.3	31.0	63.9	16.8
Ketoconazole	>100	>100	>100	>100	32.5

**Table 4:** In vitro stability study of compound **10**, FTY720, and PF-543 compared with verapamil on human, rat, and mouse liver microsomes.

Compound	Human (%)	Rat (%)	Mouse (%)
Compound **10**	70.3	10.2	44.7
FTY720	90.5	77.0	89.9
PF543	13.5	8.1	6.5
Verapamil (Reference)	14.4	–	–

## Supplementary Materials

The following are available online at https://www.mdpi.com/article/10.3390/pharmaceutics14010157/s1. Figure S1: Relative IC_50_ determination of compound **10** (A, B), and comparison of cytotoxicity of compound **10** with compound **11** and **13** (C). Figure S2: The effects of compounds in the viability of pancreatic cancer and gastric cancer cells.
