# Supplementary material for: Determining the Anticancer Activity of Sphingosine Kinase Inhibitors Containing Heteroatoms in Their Tail Structure
Source: Pharmaceutics. 2022 Jan 10;14(1):157. doi: 10.3390/pharmaceutics14010157 (PMC8779255; doi:10.3390/pharmaceutics14010157)
Supplement: Supplementary file 1 [file pharmaceutics-14-00157-s001.zip › pharmaceutics-1481642-supplementary.pdf]

pharmaceutics

Supplementary Materials

Determining the Anticancer Activity of Sphingosine Kinase Inhibitors Containing Heteroatoms in Their Tail Structure

Jitendra Shrestha [1,†], Seong Woong Kim [1,†], Su-Bin Kim [1], Yoon Sin Oh [2], Sung Hwan Ki [3], Taeho Lee [4], Sang-Bum Kim [5], Taeuk Park [6], Dong Jae Baek [1,*] and Eun-Young Park [1,*]

[1] College of Pharmacy, Mokpo National University, Muan-gun 58554, Korea; shresthasimon2011@gmail.com (J.S.); 76pey@naver.com (S.W.K.); rlatnqls0801@naver.com (S.B.K.)
[2] Department of Food and Nutrition, Eulji University, Seongnam 13135, Korea; ysoh@eulji.ac.kr
[3] College of Pharmacy, Chosun University, Gwangju 61452, Korea; shki@chosun.ac.kr
[4] College of Pharmacy, Research Institute of Pharmaceutical Sciences, Kyungpook National University, Daegu 41566, Korea; tlee@knu.ac.kr
[5] New Drug Development Center, Daegu-Gyeongbuk Medical Innovation Foundation, Daegu 41061, Korea; ksb2014@dgmif.re.kr
[6] Laboratory Animal Center, Daegu-Gyeongbuk Medical Innovation Foundation, Daegu 41061, Korea; tw3000@dgmif.re.kr
* Correspondence: dbaek@mokpo.ac.kr (D.J.B.); parkey@mokpo.ac.kr (E.Y.P.)
† These authors contributed equally to this work.

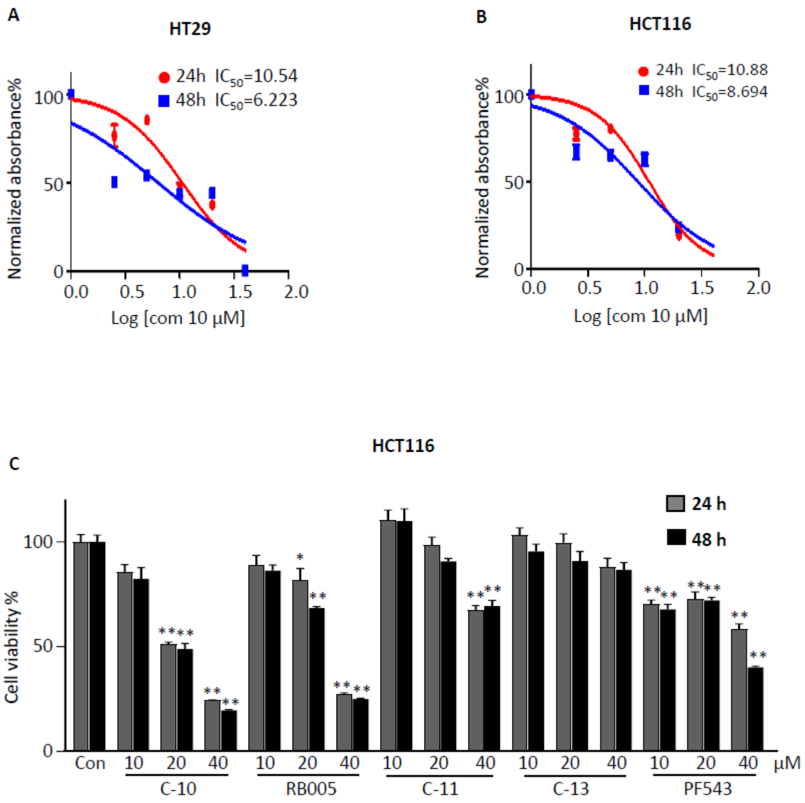

Figure S1. Relative IC50 determination of Compound 10, and comparison of cytotoxicity of compound 10 with compound 11 and 13. (**A**) HT29 and (**B**) HCT116 cells were seeded in 96 well plates and treated with 2.5-40 μM of compound 10 for 24h and 48h. Cell viability was assessed using MTT assay, and Relative IC50 was determined using Graph pad prism software. (**C**) HCT116 cells were seeded in 96 well plates and treated with 10-40 μM of compound 10, 11 and 13 for 24h and 48h. Cell viability was determined using MTT cell viability assay kit. The result is representative of three independent experiments and data are presented as mean ± SD. * $p < 0.05$, ** $p < 0.01$ compared with non-treated control group.

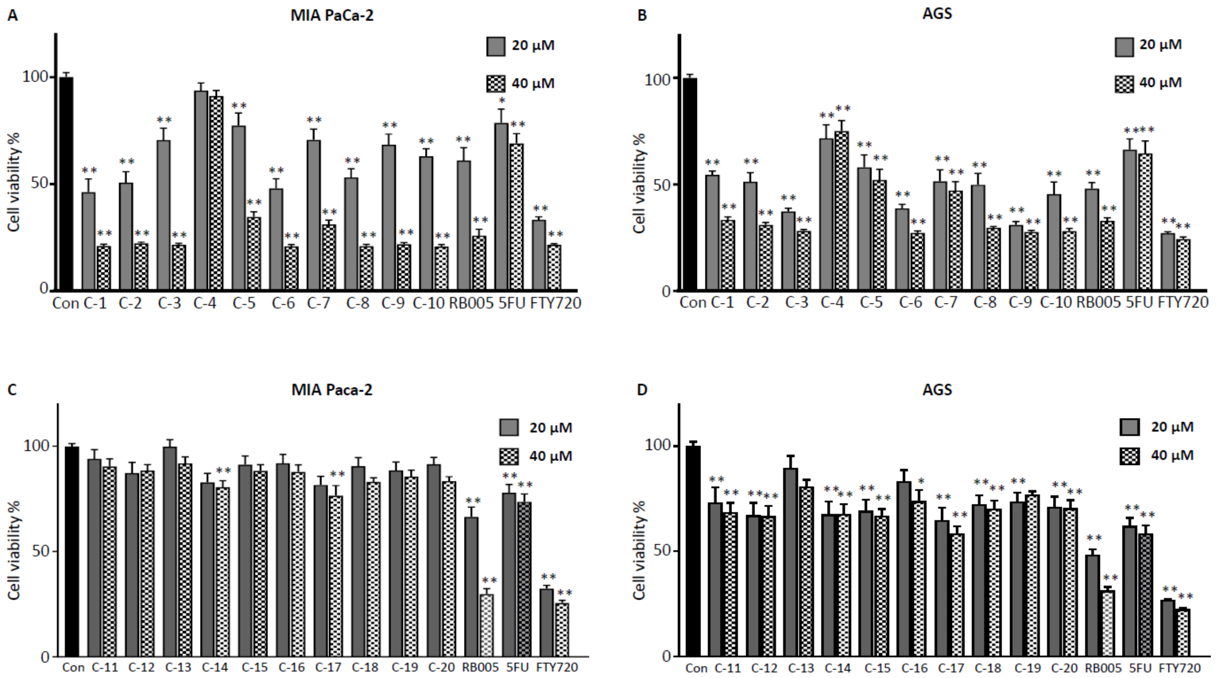

Figure S2. The effects of compounds in the viability of pancreatic cancer and gastric cancer cells. The viability of MIA Paca-2 (**A, C**) and AGS cells (**B, D**) were determined after 24 h treatment of compound (20 and 40 μM) **1**-**10** and **11**-**20** using MTT cell viability assay. Each cancer cell (3×10^3 cells/well) was seeded in 96 well plates and treated with respective compounds in desired concentrations for 24h. The results presented are mean ± SD for three individual experiments. * $p < 0.05$, ** $p < 0.01$ compared with non-treated control group.
